# Communication Signal Modulation Recognition Method Based on Multi-Feature Multi-Channel ResNet and BiLSTM Neural Network

**DOI:** 10.3390/s26051426

**Published:** 2026-02-25

**Authors:** Xi Li, Xuan Geng, Yanli Xu, Fang Cao

**Affiliations:** College of Information Engineering, Shanghai Maritime University, Shanghai 201306, China; 202330310046@stu.shmtu.edu.cn (X.L.); ylxu@shmtu.edu.cn (Y.X.); fangcao@shmtu.edu.cn (F.C.)

**Keywords:** automatic modulation recognition, deep learning, feature fusion, multichannel networks

## Abstract

To deal with the insufficient recognition accuracy of traditional signal modulation recognition methods, this paper proposes a new communication signal modulation recognition method with a deep neural network that integrates a multi-feature multi-channel ResNet and BiLSTM neural network (MF-MC ResNet-BiLSTM). By converting the original modulation data into three different vector formats, which are IQ format, AP format, and FFT format, we obtained the model inputs which contain various feature information. After inputting three types of vector signals into the multi-channel feature fusion module, the network converts these input signals into a high-dimensional feature space for feature fusion, and extracts features we need from different signal sources. Meanwhile, we designed a multi-channel model that integrates ResNet-BiLSTM to perform feature fusion, extracting key features of the modulation signal to avoid the degradation of orthogonality caused by parameter imbalance. To further enhance modulation recognition performance, an adaptive multi-head attention network was designed to extract features through weighted integration. Simulation results demonstrate that this method exhibits model generalization capabilities and good robustness. Experimental data validate that the method achieves a recognition rate of 95.67% and a recall rate of 94.56% in low signal-to-noise ratio (SNR) environments (−22 dB–2 dB), significantly outperforming existing networks like MMF(multimodal fusion), FGDNN(fusion GRU deep learning neural network), and LightMFFS(redlightweight multi-feature fusion structure).

## 1. Introduction

Automatic Modulation Recognition (AMR) is a technology that identifies the modulation type of the received signal automatically by analyzing its characteristics in the case that prior information is unknown, serving as a crucial means for monitoring and managing spectrum resources [1]. However, it usually requires a large amount of computing resources and has a high dependence on manually designed features, which limits the ability to express features [2].

Currently, most deep learning-based Automatic Modulation Classification (AMC) methods rely on one-dimensional signal information, failing to fully exploit the characteristics across different signal dimensions. To address this, multi-modal techniques have been widely adopted in the AMC field in recent years and their effectiveness in improving recognition accuracy has been confirmed. Zhang et al. [3] pointed out that fusion of diverse features can enhance the effectiveness of modulation recognition. Deng et al. [4] indicated that multi-modal feature fusion can improve the joint utilization of features to boost classification accuracy. Liu et al. [5] proposed a multi-modal technique based on contrastive learning and demonstrated its effectiveness on the RML2016.10a dataset. Sun et al. [6] introduced a method combining Gated Recurrent Units (GRU) and Convolutional Neural Networks (CNN). They utilized the IQ data and Amplitude-Phase (AP) data for feature extraction and classification, achieving excellent performance on the RML2018.01a dataset. Pathak et al. [7] proposed a parallel multi-channel learning framework to process both IQ and AP modal information, using Long Short-Term Memory (LSTM) networks to extract temporal information and fully connected layers for classification. Wang et al. [8] presented a multi-stream neural network utilizing spatial attention and multi-head attention mechanisms. This method enhanced the dynamic weight allocation capability for spatial and temporal features extracted by the CNN and Bidirectional Gated Recurrent Unit (BiGRU).

In order to address the low SNR and shortcomings of the classification of signals that are easily confused in current models, we proposes a modulation signal classification and recognition algorithm that integrates a multi-feature multi-channel ResNet and BiLSTM neural network. Firstly, the original modulated data are converted into three different vector formats as model inputs including IQ signal, AP signal and FFT signal. Secondly, a multi-channel network architecture is designed to extract multi-dimensional features of input signals. Then, the ResNet network combining depthwise separable convolutional network (DSC) is designed to optimize computational efficiency. Finally, an adaptive multi-head attention network with weighted integration extraction capabilities combining BiLSTM is designed to further improve modulated signal recognition performance. Experimental results show that this method achieves a recognition rate of 95.67% and a recall rate of 94.56% in low SNR environments (−22 dB–2 dB), significantly outperforming existing mainstream network models like MMF, FGDNN, and LightMFFS.

## 2. Model of Automatic Signal Modulation System

**Figure 1:**
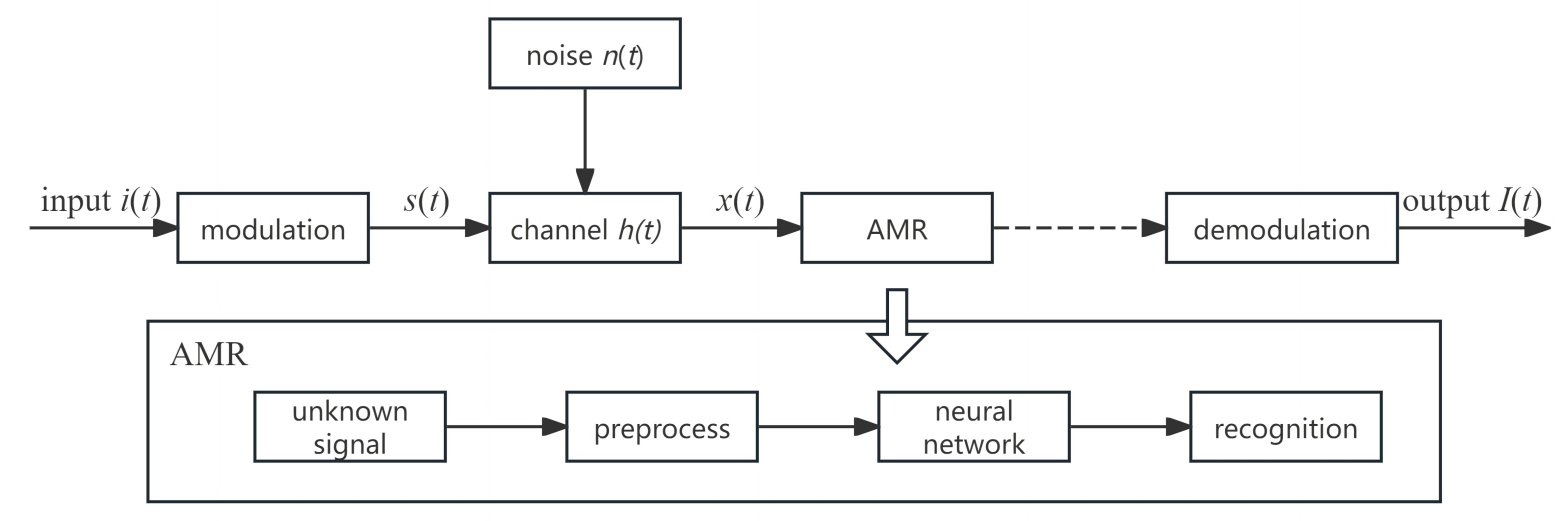
Modulation recognition system model based on deep learning.

The deep learning-based automatic modulation recognition system model [9] is shown in Figure 1.

First, the input signal i(t) is modulated to be s(t) After that, the s(t) is transmitted through channel h(t) and is affected by noise interference and channel fading n(t). The receiver receives the signal as x(t), and the received signal x(t) can be expressed by Equation (1).

(1) x(t)=s(t)∗h(t)+n(t)

where s(t) is the transmitted signal, which can be expressed as follows.

(2) $$s\left(t\right)=\sum_{k=-\infty }^{\infty }a_{k}g\left(t-kT_{s}\right)e^{j\left(2\pi f_{c}t+\theta \right)}$$

where $a_{k}$ is the sampling symbol of i(t) which is $a_{k}=i\left(kT_{s}\right)$. g(t) is the pulse shaping function. $T_{s}$ is the symbol period. $f_{c}$ is the carrier frequency, and θ is the initial phase. Since the dataset used in this paper is in IQ vector format, it can also be expressed by Equation (3).

(3) s(t)=I(t)·cos(ϕ)−Q(t)·sin(ϕ)

where I(t) is the in-phase component of s(t) and Q(t) is the quadrature component of s(t), and the phase $\phi =2\pi f_{c}t$.

The h(t) is the channel impulse response, modeled as the effect of multi-path effects and fading which is as follows.

(4) $$h\left(t\right)=\sum_{l=0}^{L-1}\gamma_{l}\delta \left(t-\tau_{l}\right)$$

where $\gamma_{l}$ and $\tau_{l}$ denote the fading coefficient and delay of the first *l* path, respectively. n(t) is additive Gaussian white noise (AWGN), obeying the Gaussian distribution $N\left(0,\sigma^{2}\right)$.

In a discrete sampling system, the received signal can usually be expressed as a sequence of in-phase/quadrature (I/Q) components in complex form.

(5) x[n]=I[n]+jQ[n],n=0,1,…,N−1

where *N* is the number of samples and I[n] and Q[n] are the discrete samples of the in-phase and quadrature components, respectively.

The processing of the received signal x(t) at the receiver is mainly divided into two stages. Firstly, the received signal is preprocessed to derive a high-quality signal representation. Secondly, deep learning algorithms such as residual networks and long short-term memory networks are utilized to achieve automatic extraction of signal features and prediction of the modulation type of the signal.

## 3. Modulated Signal Classification and Recognition Algorithm Based on Fusion Multi-Feature Multi-Channel ResNet and BiLSTM Network

### 3.1. Network Model Design

We propose a modulated signal classification and recognition algorithm based on the fusion multi-feature multi-channel ResNet and BiLSTM network (mf-mc ResNet-BiLSTM) and automatic modulation recognition model, and the designed network model is as follows.

Firstly, the training dataset is produced, where the original modulated signal is performed as IQ vectors and then the IQ vectors are converted into AP and FFT signals respectively. After inputting three types of vector signals into the multi-channel feature fusion module, the processing module converts these input signals into a high-dimensional feature space for feature fusion and extracts useful features from different signal sources [10].

Then we designed the residual neural network (ResNet) based on depthwise separable convolution, which serves as a lightweight module to process spatial sequences which can further accelerate the training process. After that, we designed the feature extraction and fusion network. We proposed the model which combines DSC with bidirectional long short-term memory networks (BiLSTM) to extract temporal features of the signals. Finally, we designed a self-attention mechanism, which can focus on the temporal feature extraction in BiLSTM. To deal with the problem that the self-attention mechanism has low expressive ability, we designed the multi-head attention mechanism (MHA) to increase the model’s expressive ability and capture long-range dependence between features, which achieved the goal of network optimization.

**Figure 2:**
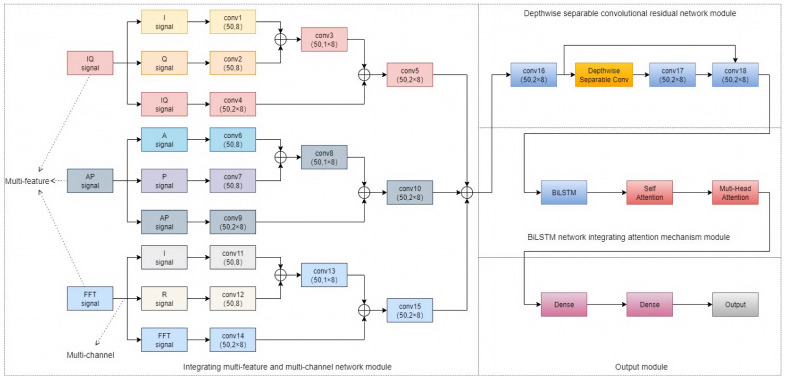
Network model.

Through the DNN network, the extracted temporal features are input into the data in a one-dimensional format, and after two full-connectivity layer dimensionality reductions, the final output is the recognition results of signal modulation categories. The category predicted by the neural network is $\hat{y}$, and the cross-entropy function is used to measure the difference between the predicted category $\hat{y}$ and the real category *y*. The parameter update is carried out with the goal of minimizing the cross-entropy loss function and the expression of the loss function.

(6) $$L=-\frac{1}{N}\sum_{i=1}^{N}\left[y^{i}\ln{\hat{y}}^{i}+\left(1-\hat{y}\right)\ln\left(1-{\hat{y}}^{i}\right)\right]$$

where *N* is the total number of samples, $y^{i}$ is the true category label of the *i*th sample, and ${\hat{y}}^{i}$ is the predicted value of the *i*th sample given by the model. The overall architecture of the algorithm is shown in Figure 2.

### 3.2. Input Data Design

Currently, the deep learning schemes mainly use single-vector signal inputs, such as IQ signals or frequency domain FFT signals. But the characterization ability of IQ signals and FFT signals significantly decreases in low SNR environments, and the recognition accuracy of the model is greatly reduced under noise interference.

In this paper, we propose a fusion model using IQ vectors, AP vectors and FFT vectors to formulate the input data. In this model, the IQ vectors retain the original temporal characteristics. AP vectors strengthen the temporal and spatial correlation between amplitude and phase in the form of polar coordinates, and FFT vectors provide the global spectral features through the extraction of the frequency domain.

#### 3.2.1. IQ Vector

The IQ vector $X^{IQ}$ is obtained by multiplying the I-channel signals I(t) with cosϕ and the Q-channel signals Q(t) with sinϕ. Then, we superimpose them to get the modulation signal s(t), where the in-phase component is $x^{I}$ and the quadrature component is $x^{Q}$, denoted as follows.

(7) $$x^{IQ}=\left[\begin{array}{c} x^{I^{r}}\\ x^{Q^{r}} \end{array}\right]$$

#### 3.2.2. AP Vector

The AP vector $x^{AP}$ refers to the joint amplitude-phase characterization, which is converted by the IQ vector to get the amplitude $x^{A}$ and phase data $x^{P}$.

(8) $$x^{AP}=\left[\begin{array}{c} x^{A^{T}}\\ x^{P^{T}} \end{array}\right]$$

Its calculation equation is as follows.

(9) $$\left\{\begin{array}{c} x_{n}^{p}=\arctan\left(\frac{x_{n}^{Q}}{x_{n}^{I}}\right),n=0,1\ldots ,N-1\\ x_{n}^{A}=\sqrt{{\left(x_{n}^{I}\right)}^{2}+{\left(x_{n}^{Q}\right)}^{2}},n=0,1\ldots ,N-1 \end{array}\right.$$

where $x_{n}^{P}$ is the phase data of AP vector and $x_{n}^{A}$ is the amplitude data of AP vector. After the transformation, the vector formats of $x_{n}^{P}$ and $x_{n}^{A}$ are the same as the IQ vectors before the transformation, which can be uniformly input into the same model structure without additional operations, greatly simplifying the complexity of the model.

#### 3.2.3. FFT Vector

The FFT vector $x^{FFT}$ is the mapping of the IQ vector from the time domain to the frequency domain as shown in Equation (10).

(10) $$x^{FFT}=\left[\begin{array}{c} x^{FFT-Re^{r}}\\ x^{FFT-Im^{r}} \end{array}\right]=\left[\begin{array}{c} Re\{FFT[r(k)]\}\\ Im\{FFT[r(k)]\} \end{array}\right]$$

First of all, r(n) is a complex signal representation of the IQ channel.

(11) $$r_{n}=x_{n}^{I}+j^{*}x_{n}^{Q}$$

The discrete Fourier transform is then applied to the complex signal r(n), which can be written as follows.

(12) $$x^{FFT}\left(k\right)=\frac{1}{N}\sum_{i=0}^{N}r_{n}e^{-2j\pi (k)ki/N}$$

Finally, after obtaining the spectral signal $x^{FFT}$, we divided the spectral signal into two channels, real and imaginary, as the input to the neural network.

### 3.3. Multi-Feature and Multi-Channel Network

**Figure 3:**
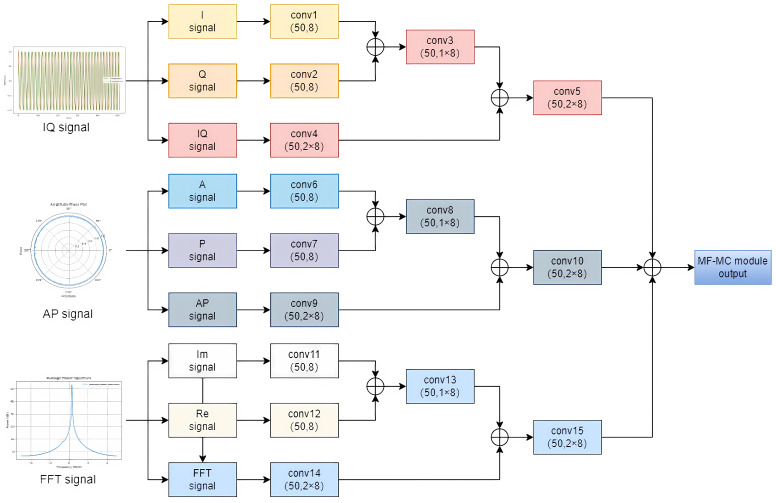
Multi-feature and multi-channel network architecture diagram.

In order to fully utilize the multi-dimensional information of the signal and improve the accuracy and robustness of the modulation recognition, we designed and integrated the multi-feature network (MF) and the multi-channel network (MC) [11]. MF refers to the strategy which employs three formats as inputs, including the raw IQ signal, amplitude-phase (AP) vector, and frequency vector, to extract signal features. MC refers to the parallel processing of signals through multiple independent channels. Each channel can extract different features of the signal and then fuse these features to enhance recognition. The multi-feature and multi-channel network architecture is shown in Figure 3.

We take the FM signal as an example, as shown in Figure 3. Firstly, in the MF module, three vector formats of FM signals are input, which contain different amplitude, phase, and frequency domain information.

Secondly, we put the fused signals into the MC module. The IQ channel uses M×N as the main channel and splits the IQ data into in-phase and quadrature components to obtain the I and Q channels in the format of 1/2×M×N. The I and Q channels are used as auxiliary channels, which can be effectively supplemented with features for the main channel. In this way, the main channel and the auxiliary channel complement each other, reducing the possibility of missing features and making the feature characterization more stable and diversified. The multi-channel network parameters are shown in Table 1. Since the AP, FFT signals and IQ signals have similar processing methods, we take IQ data as an example to describe in detail.

Finally, we put the fused signals into the MC module. This module converts different types of input signals into a high-dimensional feature space and performs feature fusion, making the network able to extract useful features from different signal sources and combine the features to improve classification performance. After feature fusion, the system classifies three different vector and calculates the corresponding probabilities for each class.

### 3.4. Depth Separable Convolution Based Residual Network and BiLSTM Incorporating Attention Mechanism

#### 3.4.1. Depth Separable Convolution Based Residual Network

In order to make the network learn deeper feature representations and effectively deal with gradient vanishing in deep networks, we designed a network combining Depthwise Separable Convolution (DSC) and Residual Network (Residual Network based on Depthwise Separable Convolution, RNDSC). This module can reduce the size of the model and the number of parameters to improve the speed of the model and the computational efficiency at the same time.

The DSC is an important technique used in convolutional neural networks to optimize computational efficiency, which can be decomposed into two steps including of depthwise convolution and pointwise convolution. For DSC, depthwise convolution is used to extract spatial features, while pointwise convolution is employed to extract channel features. DSC performs grouped convolution in feature dimensions, applying independent depthwise convolution to each channel, and then aggregates all channels using a 1 × 1 convolution before the output. The number of parameters and computation amount is the sum of the number of parameters and computation amount of the two steps of depthwise convolution and pointwise convolution. The parameter ratio and computation complexity ratio of the conventional and DSC are as follows.

(13) $$\frac{N_{DSC}}{N_{Conv}}=\frac{C_{DSC}}{C_{Conv}}=\frac{1}{N}+\frac{1}{D_{K}\times D_{K}}$$

where $N_{DSC}$ and $N_{Conv}$ are the parameter count of DSC and conventional convolution. $C_{DSC}$ and $C_{Conv}$ are the computation complexity of DSC and conventional convolution. *N* is the number of convolution kernels and $D_{K}$ is the size of the convolution kernel. From Equation (13), the parameter and computational complexity of DSC are much smaller than that of Conv. Therefore, DSC is adopted to achieve a lightweight network structure.

**Figure 4:**
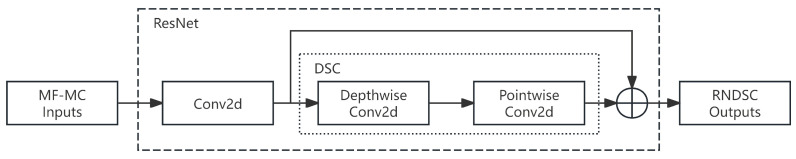
Residual network structure based on depthwise separable convolution.

Meanwhile, we designed a new ResNet based on the DSC which is shown in Figure 4. Firstly, the network extracts features through two-dimensional convolution on MF-MC input features. Secondly, the output features is processed by depthwise convolution, and then the pointwise convolution is employed for dimensionality reduction and fusion. Finally, the fused feature is output by RNDSC. This structure enables the model to learn the features of input data and enhances the robustness and generalization ability of the model.

#### 3.4.2. BiLSTM Incorporating Attention Mechanism

**Figure 5:**
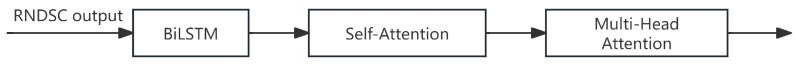
BiLSTM network integrating attention mechanism.

The features which are the output of ResNet need to be processed in the temporal feature part, so we proposed the BiLSTM with fusion attention mechanism which connects Self-Attention and Multi-Head Attention. We named this module BSM, and its structural diagram is shown in Figure 5.

The feature sequence generated by DSC is shown as follows.

(14) $$Z=\left\{z_{1},z_{2},\ldots ,z_{T}\right\}$$

*Z* is the vector of length *T* and $z_{T}$ is the *T*th valued feature vector. After inputting *Z* into a BiLSTM recurrent network, we used two LSTM networks to scan along the forward and backward directions of the sequence. The hidden states of the two LSTM networks will be concatenated at each position, where ${\vec{v}}_{t}$ denotes the forward output of the hidden layer unit at the time of *t*. ${\vec{g}}^{\mathrm{LSTM}}$ denotes the gate control of LSTM in forward propagation. The output is controlled by the memory unit $c_{t-1}$ at the time of t−1 and the forward output ${\vec{v}}_{t-1}$ at the time of t−1. Similarly, ${\overset{\leftarrow }{v}}_{t}$ denotes the backward output of the hidden layer unit at the time of *t* and the parameter $W_{t}$ in the forward and backward LSTM network is shared. Then the output of the hidden layer unit at the time of *t* can be expressed as a cascade of the hidden states of the forward and backward LSTM network, and the long-term dependency of the feature sequences can be better captured and prevent information loss, which is expressed as follows.

(15) $$\left\{\begin{array}{c} c_{t},{\vec{v}}_{t}={\vec{g}}^{LSTM}\left(c_{t-1},{\vec{v}}_{t-1},W_{t}\right)\\ c_{t},{\overset{\leftarrow }{v}}_{t}={\vec{g}}^{LSTM}\left(c_{t-1},{\overset{\leftarrow }{v}}_{t-1},W_{t}\right)\\ v_{t}={\vec{v}}_{t}⊕{\overset{\leftarrow }{v}}_{t} \end{array}\right.$$

Then, we calculated the attention probability distribution value *E* determined by the output *V* from the BiLSTM, which is represented as follows.

(16) E=U∗(tanh(W∗V+b))

where *U*, *W* is the weight coefficient and *b* is the bias coefficient. The output *V* from the BiLSTM denotes $V=\left\{v_{1},v_{2},\ldots v_{t},\ldots v_{T}\right\}$.

After training to obtain the weight vector $a_{t}$ corresponding to each output vector $v_{t}$, the weights are applied to the hidden states output by BiLSTM. Then the output of the self-attention layer *c* is a weighted summation of the weights, which is represented as follows.

(17) $$c=\alpha H=\sum_{t=1}^{T}\alpha_{t}\cdot v_{t}$$

where α is the regularized weight of attention probability distribution value *E*, and its formula can be expressed as α=softmax(E).

Then, we put the output of the self-attention mechanism *c* into the multi-head attention module. The multi-head attention mechanism is set as *u* head, represented by the attention mechanism unit ${\mathrm{head}}_{i}$, and the attention mechanism is performed from within different attention units to learn different relevant features, where the output of the *i*th attention unit is represented as follows.

(18) $${\mathrm{head}}_{i}=\beta_{i}c=\sum_{t=1}^{T}\beta_{it}\cdot c_{t}$$

where $\beta_{i}$ is the regularized weight of the attention unit ${\mathrm{head}}_{i}$ and the sum of the weights of $\beta_{it}$ under the attention mechanism unit is 1.

The output feature result ${\mathrm{head}}_{i}$ is concatenated and then linearly transformed to get the final output of the attention expressed as follows.

(19) $$\mathrm{Output}=\left[\begin{array}{c} {\mathrm{head}}_{1}\\ {\mathrm{head}}_{2}\\ \cdot \\ \cdot \\ \cdot \\ {\mathrm{head}}_{u} \end{array}\right]W^{\circ }$$

**Figure 6:**
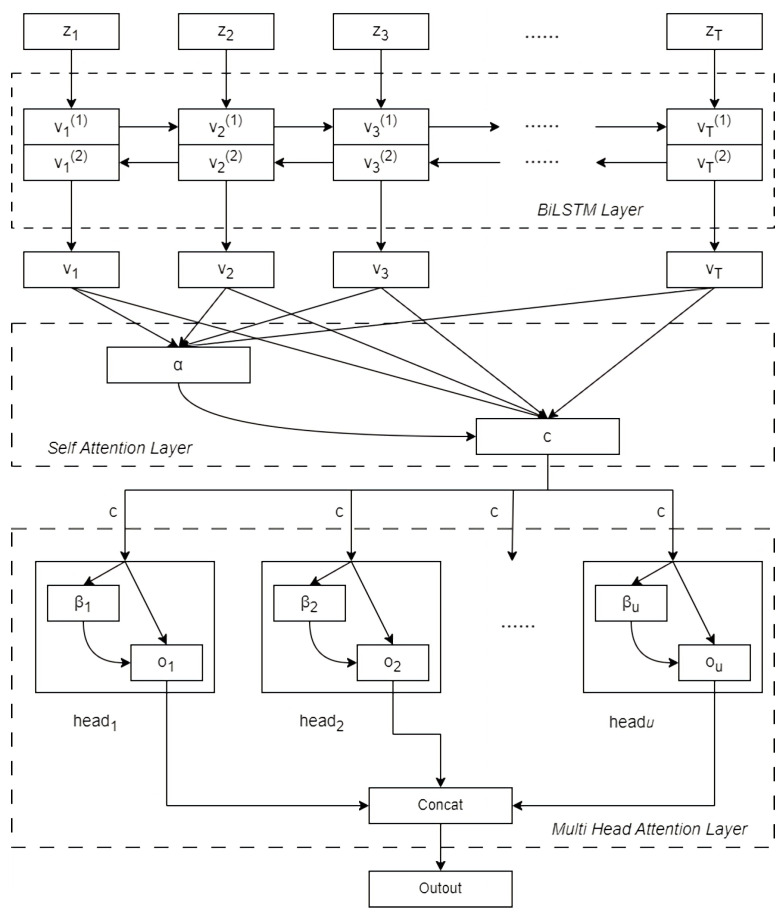
Algorithm diagram of BiLSTM network structure integrating attention mechanism.

The output of the attention layer is passed into the dense layer twice to realize the dimensionality reduction and generate a k-dimensional matrix, and finally the discrimination type of the modulated recognized signal is obtained by argmax operation. The detailed process of the bidirectional long short-term memory network structure algorithm incorporating the attention mechanism is shown in Figure 6.

### 3.5. Model Training

The DSC can simplify the training and the ResNet can solve the gradient vanishing [12] or gradient bursting. Meanwhile, the BSM can comprehensively capture the features. Therefore, the modulation recognition accuracy of the signal can be significantly improved by combining these modules.

In order to speed up the training process, we adopt the mini-batch approach to process the data. That means the training set is divided into *n* batches and each batch contains $n_{m}$ training samples. During the training process, one batch is selected at one time. The loss function for constructing the model is shown as follows.

(20) $$\mathrm{Loss}\left(\theta \right)=-\frac{1}{n_{m}}\sum_{i=1}^{n_{m}}y_{i}\ln{\hat{y}}_{i}+\left(1-y_{i}\right)\ln\left(1-y_{i}\right)$$

The optimization algorithm is selected as the Adam optimizer, and the parameter updates follow a scheduling function that dynamically adjusts the learning rate. We summarize the training process as shown below.

(1) Initialize the network model parameters and set the learning rate.
(2) Randomly divide the training samples into *n* batches.
(3) Randomly select a batch $n_{m}$.
(4) Input the sample s(t) into the multichannel network in multi-vector format.
(5) Use RNDSC to simplify the training. Then use BSM to train the network on the feature sequence *Z* which is the output of the multi-feature multi-channel network. Finally, we use Output as the output.
(6) Use Softmax to obtain the predictive probability distribution of the model $y_{i}$.
(7) Calculate the loss function by substituting the value of $y_{i}$ into Equation (6)
(8) Update the parameters using Adam’s algorithm θ.
(9) Repeat steps (2) to (9) until the loss function converges.

## 4. Experimental Results and Discussion

### 4.1. Experimental Setup and Dataset

In this experiment, Python3.7 is used as the development language, and TensorFlow-GPU 2.5.0 is used as the computing framework, which utilizes Keras 2.5.0 as the built-in high-level neural network API. CUDA8.0 is used for computing acceleration. The CPU used in the experiment is Intel (R) Xeon (R) Gold 5418Y, and the GPU is RTX 4090.

The RML2018.01a dataset was used as the input data for this experiment [13]. This dataset is generated by GNU Radio over a complex channel and contains the main impairments of wireless signals in a real-world environment, as well as 24 signal modulation types with a sampling rate of 64 kHz and a maximum sampling rate offset of 50 Hz. Each signal consists of 1024 samples, including a total of 2,555,904 signal samples with SNR ranging from −20 dB to 30 dB, 2 dB intervals. The specific parameters are shown in Table 2.

The simulation experiments are partially constructed using the Keras in Python. To optimize the network parameters effectively, the simulation uses the Adam optimizer. The dataset is divided into a training set, validation set, and test set, in which the training set accounts for 64%, the validation set accounts for 16%, and the test set accounts for 20%, and such a division is helpful for cross-validation and performance evaluation during model training. The specific parameter settings of the model are shown in Table 3.

### 4.2. Experiments Results for Multi-Feature Inputs

#### 4.2.1. Confusion Matrix Analysis

**Figure 7:**
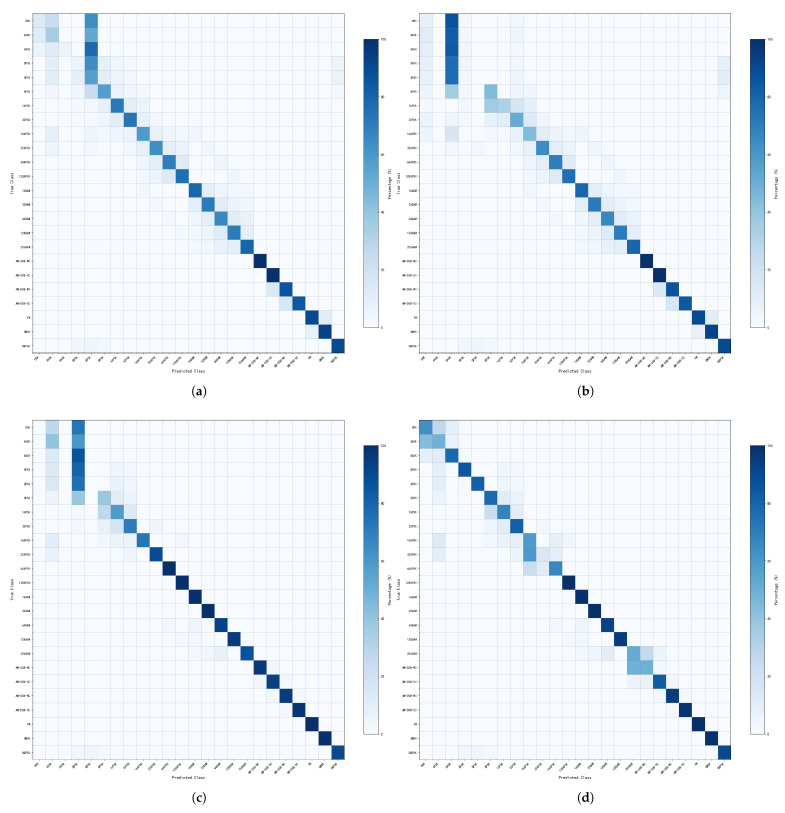
Confusion matrix of various vector signals input into the network. (**a**) IQ vector format input. (**b**) AP vector format input. (**c**) FFT vector format input. (**d**) Multi-vector format fusion input.

In this study, several different types of signal inputs are used, including IQ vector, AP vector and FFT vector. The effect of recognizing modulated signals under different types of signal inputs is further verified by using them as single-vector signal inputs and multi-vector signal inputs respectively. Figure 7 gives the confusion matrix of all signals under the inputs of IQ vector, AP vector, FFT vector and multi-vector inputs. The SNR range is −20–30 dB.

As can be seen from Figure 7, the diagonal lines of the confusion matrix of QAM, AM-SSB, AM-DSB, FM, GMSK, and OQPSK are relatively clear for both the single-vector and multi-vector signal inputs, which indicates that they can distinguish various modulated signals more accurately. For signals such as ASK, BPSK, QPSK, PSK, and APSK, the confusion matrices of the single-vector signal inputs show more misclassification cases, and the classification boundaries are more blurred. In contrast, the confusion matrices for multi-vector signal inputs have relatively clear diagonal lines in these signals, indicating that they are able to distinguish various types of modulated signals more accurately.

#### 4.2.2. Noise Resistance Analysis

**Figure 8:**
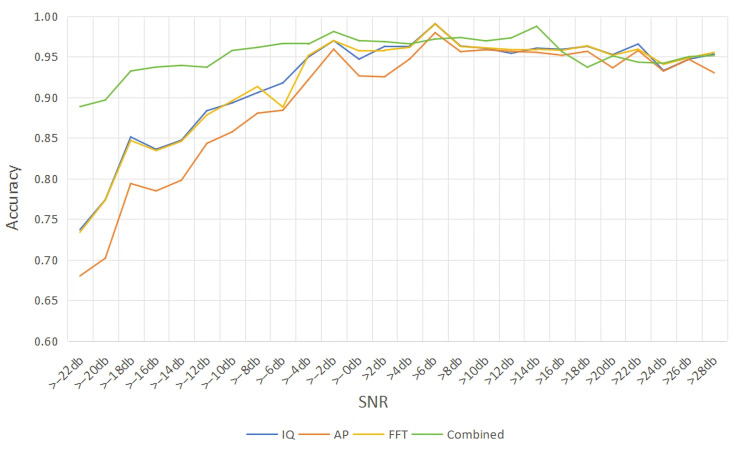
Accuracy comparison of single vector signal and multi-vector signal.

To explore the recognition performance changes of the multi-vector signal input in the environment of different SNRs. We fused the original signals of different SNR and obtained the signals with a certain range of SNRs to replace the single SNR signal as the input in order to better suit the actual environment. We take the >24 dB in Figure 8 as an example, which refers to the signal input which is composed of all the signals in the 26 dB, 28 dB, and 30 dB SNR for the signals. Figure 8 gives the accuracy of the modulated signal under the SNR with −20–30 dB for the single-vector signal input and the multi-vector signal input.

As shown in Figure 8, before >6 dB, the FFT vector signal has the lowest accuracy at the beginning but has the fastest increase in accuracy. The IQ vector signal and AP vector signal have lower and closer accuracy, and the increase in accuracy is also more rapid and smooth. The multi-vector signal inputs investigated in this paper have higher accuracy than single-vector signal inputs before >6 dB. Meanwhile, the multi-vector signal inputs can achieve an accuracy of 95% after >−10 dB. After >6 dB, the accuracy of single-vector and multi-vector signal inputs is not much different and the trend is relatively smooth; both can reach about 95%.

### 4.3. Multi-Channel Network Performance Analysis

**Figure 9:**
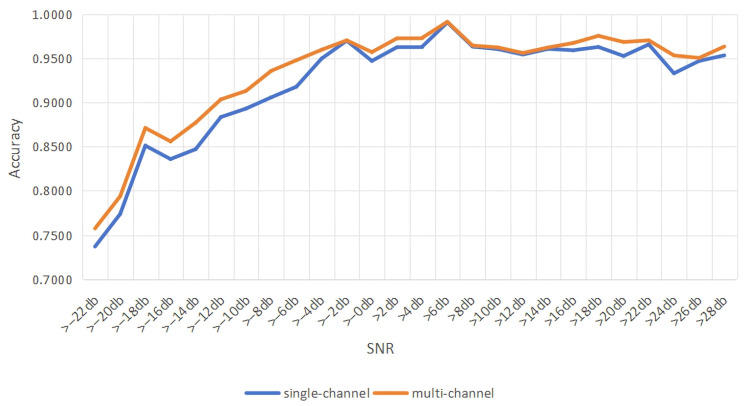
Accuracy comparison of single-channel and multi-channel networks.

To further verify the improved performance of multi-channel networks [14] in recognizing modulated signals, we take the IQ vector signals as an example to compare the accuracy obtained by multi-channel and single-channel networks, resectively. Firstly, we use the original IQ signal as a single-channel input model. Then, we input the I channel, Q channel, and IQ channel into the model at the same time for the experimental simulation and comparison. The results of the simulation are shown in Figure 9.

As shown in Figure 9, the multi-channel network has an improvement in accuracy compared to the single-channel network [15]. Since dividing the IQ signal into I-component, Q-component and IQ-component can make it easier for the model to identify and analyze the features of each component, the multi-channel network can extract the features more adequately for >0 dB before. Meanwhile, the FFT and AP signals have a similar principle to the IQ signal and also have a significant improvement in accuracy.

### 4.4. Performance Analysis of BiLSTM Networks with Attention Mechanisms

**Figure 10:**
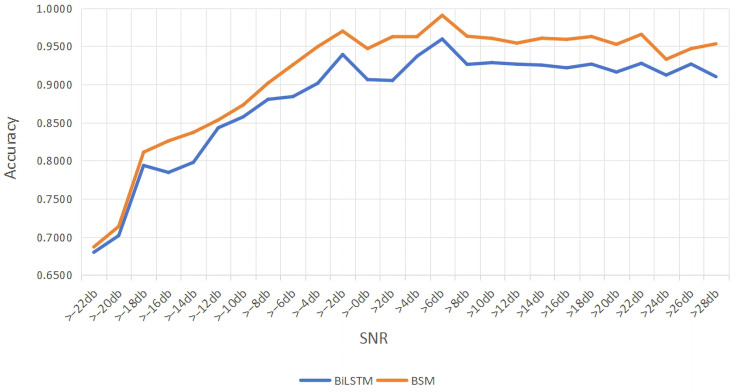
Accuary comparison of accuracy between BiLSTM and BSM.

In order to verify the effectiveness of the fused multi-head attention mechanism, we designed a comparison experiment after the RNDSC module. We verify the necessity of incorporating a fusion multi-head attention mechanism [16] by comparing the changes in recognition accuracy before and after BSM module. The simulation results are shown in Figure 10.

From Figure 10, it can be seen that the BiLSTM model has ability in extracting signal features. But due to its relatively simple structure, it cannot fully capture the features of complex signals which are not easy to extract, and its recognition accuracy is only 93%. Introducing the attention mechanism and the adaptive mechanism with multiple heads into BiLSTM significantly enhances the model’s ability to extract important features. Meanwhile, BSM can better handle complex signals, and its recognition accuracy is improved to 95.5%.

### 4.5. Number of Parameters Analysis

In this section, we discuss the number of parameters of each module, which is shown in Table 4.

As can be seen in Table 4, the BiLSTM serves as the backbone module, accounting for 48.1% of the total with 0.25M parameters, and reflecting the high computing cost required for modeling complex bidirectional temporal dynamics. The Depthwise Separable Convolutional Residual Network (DSC-ResNet) contributes 28.8% with 0.15M parameters, and its relatively economical parameter consumption validates the efficiency of modern lightweight convolutional designs in feature refinement tasks. Meanwhile, the multi-head attention mechanism achieves dynamic selection and enhancement of critical information segments at a minimal cost of only 0.07M parameters (13.5%). Overall, The model places more than three-quarters of its parameters (76.9%) in the BiLSTM and DSC-ResNet modules. These components are responsible for fundamental feature extraction and temporal modeling, giving the system a solid and capable foundation. Simultaneously, it utilizes the attention mechanism as a lightweight and critical component. Ultimately, a hierarchical and focused high-efficiency recognition system was constructed with a total parameter count of 0.52M.

### 4.6. Ablation Study

In order to verify the performance of the MC-MF-RNDSC-BSM which is proposed in this paper, the experiments are conducted to combine the schemes of several modules. These modules include the following:

Module1: Multi-feature Fusion Preprocessing Module;

Module2: Multi-Channel Network Module;

Module3: Depth Separable Convolution-based Residual Network Module;

Module4: Bi-directional Long Short-Term Memory Network incorporating Attention Mechanism.

The classification recognition rates of the different methods are derived and the results are shown in Table 5. In this table, Method1 is Baseline with Module1. Method2 is Baseline with Module1 and Module2. Method3 is Baseline with Module1, Module2 and Module3. Ours contains all modules.

As can be seen in Table 5, the Baseline cannot fully capture the features of complex signals, and its recognition accuracy is only 88.33%. After the introduction of the multi-feature fusion preprocessing module and the multi-channel network module, the ability of the model to extract important features is significantly enhanced so that it can better handle complex signals, and the recognition accuracy is increased to 92.91% and the recall rate is also increased to 92.32%.

When the fusion multi-feature multi-channel network is combined with the residual network based on depth-separable convolution, the gradient problem is avoided by simplifying the model through parameter reduction and combining it with the residual network to efficiently transfer the gradient. This results in a recognition accuracy of 93.43% and a recall of 92.59%.

Finally, the combination of fused multi-feature multi-channel ResNet and BiLSTM neural network proposed in this paper can comprehensively capture the features and improve the accuracy rate to 95.67%, which is an improvement of 2.24%, and the recall rate can also reach 94.56%.

### 4.7. Training and Validation Performance

**Figure 11:**
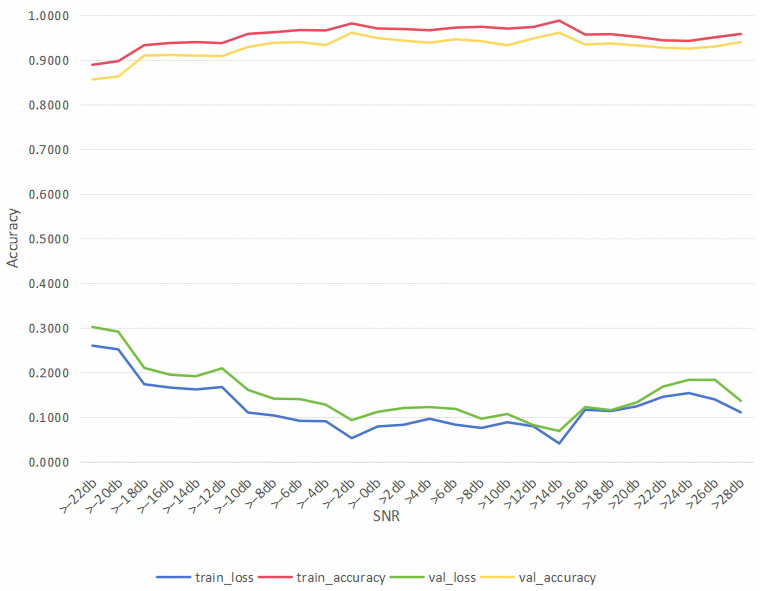
Accuracy of training and validation .

To validate whether overfitting is present, we give the training and validation performance in the Figure 11.

As shown in the Figure 11, the training and validation losses decrease simultaneously and gradually converge. Meanwhile, the training and validation accuracies improve in parallel and then stabilize, with only a small gap of approximately 3–5% between them. This limited difference indicates balanced learning behavior and good generalization performance, suggesting that no evident overfitting occurs.

### 4.8. Comparison Experiments

**Figure 12:**
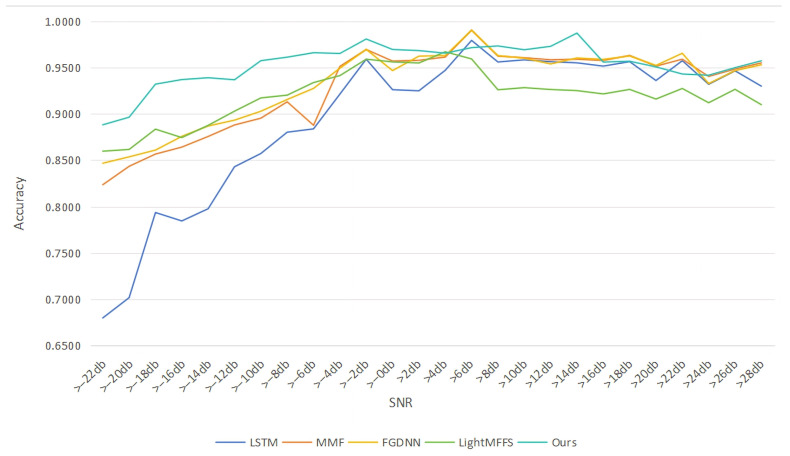
Accuracy Comparison of different methods.

To validate the performance of our model, we conducted comparision experiments with LSTM, MMF (multimodal fusion) [17], FGDNN (fusion GRU deep learning neural network) [6] and LightMFFS (lightweight multi-feature fusion structure) [18]. The accuracy of signal recognition is shown in the Figure 12.

As shown in Figure 12, before >2 dB, the accuracy of the network which we proposed has a significant improvement compared to the previous models because our network can fully extract the complex features. After >8 dB, the average precision of our network can also remain at a high level compared to the previous models. Overall, our network has significant advantages compared to previous networks.

## 5. Conclusions

In order to solve the problems of the insufficient feature expression of modulated signals and the poor effect of traditional neural networks on the classification of confusing signals, we proposed a modulated signal classification algorithm based on a multi-feature multi-channel network. By fully exploiting the interaction and diversity among different features, the richness of the dataset is enhanced and the problem of insufficient feature expression of modulated signals is effectively solved. Meanwhile, a multi-channel network structure is designed to optimize the feature extraction process for the confusing characteristics between signals, which solves the problem of the poor classification of confusing signals by traditional neural networks. In order to deal with the feature extraction inflation triggered by the increase in signal samples, we adopted a multi-head attention network to optimize the feature extraction and processing through the attention system, which ensures the high efficiency and accuracy of the model. Experimental results show that this algorithm excels in classification performance and significantly improves the accuracy and robustness of modulated signal classification. Its accuracy can reach 95.67% and the recall rate can reach 94.56%. Additionally, its accuracy has a significant improvement in low SNR environments (−22 dB–2 dB).

## Figures and Tables

**Table 1:** Multi-channel network parameters.

Neural Network Layer	Output Data Format	Parameters Count	Fully Connected Layer
I signal (Input Layer)	(batch_size, *N*, M/2)	-	-
Q signal (Input Layer)	(batch_size, *N*, M/2)	-	-
IQ signal (Input Layer)	(batch_size, *M*, *N*, M/2)	-	-
Conv1	(batch_size, *N*, 50)	450	I signal
Conv2	(batch_size, *N*, 50)	450	Q signal
Reshape1	(batch_size, M/2, *N*, 50)	-	Conv1
Reshape2	(batch_size, M/2, *N*, 50)	-	Conv2
Concatenate1	(batch_size, *M*, *N*, 50)	-	[Reshape1, Reshape2]
Conv3	(batch_size, *M*, *N*, 50)	20, 050	Concatenate1
Conv4	(batch_size, *M*, *N*, 50)	20, 050	IQ signal
Concatenate2	(batch_size, *M*, *N*, 100)	-	[Conv4, Conv3]
Conv5	(batch_size, *M*, *N*, 100)	100, 100	Concatenate2

Note: In this paper, M=2 and N=1024, batch_size ∈[64,512].

**Table 2:** RadioML 2018.01a parameters.

Dataset	RadioML 2018.01a
Data generation source	GNU Radio
Modulation	OOK, 4ASK, 8ASK, BPSK, QPSK, 8PSK, 16PSK, 32PSK.
	16APSK, 32APSK, 64APSK, 128APSK, 16QAM, 32QAM,
	64QAM, 128QAM, 256QAM, AM-SSB-WC, AM-SSB-SC,
	AM-DSB-WC, AM-DSB-SC, FM, GMSK, OQPSK
Signal-to-noise ratio range	[−20 dB, 30 dB], Interval 2 dB
Sample size	2 channels × 1024 × I/Q pairs
Total sample size	2,555,940
Sample format	IQ sample, [1024, 2].
Channel environment	Carrier frequency offset,
	symbol rate offset,
	delay, thermal noise

**Table 3:** Model parameter settings.

Parameter	Value
Dataset partitioning	Training set 64%, validation set 16%, testing set 20%
Loss function	Cross entropy function
Optimizer algorithm	Adam algorithm
Batch size	128
Learning rate	0.001
Number of iterations	56

**Table 4:** Number of parameters.

Processing Stage	Add-Parameter (M)	Cum-Parameter (M)	Proportion
+DSC-ResNet	0.15	0.15	28.8%
+BiLSTM	0.25	0.40	48.1%
+Attention	0.07	0.47	13.5%
+Connection	0.05	0.05	9.6%
Total	0.52	0.52	100%

**Table 5:** Performance comparison of ablation experomental results.

Dataset	Methods	Module1	Module2	Module3	Module4	Precision	Recall
RML2018.01a	Baseline					**88.33**	**86.62**
	Method1	✔				**92.26**	**90.13**
	Method2	✔	✔			**92.91**	**92.32**
	Method3	✔	✔	✔		**93.43**	**92.59**
	ours	✔	✔	✔	✔	**95.67**	**94.56**

Note: All the scores are described in percentages (\%). The bold data indicate the best experimental results on the dataset.

## Data Availability

The data that support the findings of this study are available from the corresponding author upon reasonable request.
